# Street lighting has detrimental impacts on local insect populations

**DOI:** 10.1126/sciadv.abi8322

**Published:** 2021-08-25

**Authors:** Douglas H. Boyes, Darren M. Evans, Richard Fox, Mark S. Parsons, Michael J. O. Pocock

**Affiliations:** 1 UK Centre for Ecology & Hydrology, Wallingford OX10 8BB, UK.; 2 School of Natural and Environmental Sciences, Newcastle University, Newcastle upon Tyne NE1 7RU, UK.; 3 Butterfly Conservation, Wareham, Dorset BH20 5QP, UK.

## Abstract

Reported declines in insect populations have sparked global concern, with artificial light at night (ALAN) identified as a potential contributing factor. Despite strong evidence that lighting disrupts a range of insect behaviors, the empirical evidence that ALAN diminishes wild insect abundance is limited. Using a matched-pairs design, we found that street lighting strongly reduced moth caterpillar abundance compared with unlit sites (47% reduction in hedgerows and 33% reduction in grass margins) and affected caterpillar development. A separate experiment in habitats with no history of lighting revealed that ALAN disrupted the feeding behavior of nocturnal caterpillars. Negative impacts were more pronounced under white light-emitting diode (LED) street lights compared to conventional yellow sodium lamps. This indicates that ALAN and the ongoing shift toward white LEDs (i.e., narrow- to broad-spectrum lighting) will have substantial consequences for insect populations and ecosystem processes.

## Teaser

Direct evidence shows that artificial light at night reduces caterpillar abundance and disrupts larval behavior and development.

## INTRODUCTION

There is growing evidence that some terrestrial insect populations have declined during recent decades (*1*–*3*), raising concerns about the future functioning of ecosystems (*4*–*7*). Of the more species-rich insect groups, moths (Lepidoptera) are the best studied, with significant population declines being reported in parts of Europe (*8*–*11*). Moths are functionally important for terrestrial ecosystems, including as pollinators, prey for both vertebrates (e.g., birds and bats) and invertebrates (e.g., spiders and social wasps), and hosts for parasitoids (*12*–*18*), and thus, these changes are expected to have substantial cascading consequences for ecosystems (*8*, *19*, *20*).

Artificial light at night (ALAN) is an increasingly recognized threat to biodiversity and ecosystem processes (*21*–*23*) and has recently been proposed as a driver of insect declines (*24*, *25*). Night lighting has wide-ranging negative effects on insects across their life cycles, including inhibiting adult activity, increased predation, and disrupted reproduction [for reviews, see (*12*, *26*, *27*)]. Several high-profile studies have highlighted the impacts of ALAN on insect pollination (*28*–*32*). Yet, it remains unclear whether the effects of ALAN are predominately disruptive impacts on the behavior of individuals or whether ALAN is actively diminishing the populations of pollinators and insect populations more broadly (*26*, *33*).

Light pollution is increasing globally (*34*) and encroaching on biodiversity hot spots (*35*). At the same time, the spectral composition of outdoor lighting is rapidly changing, with broad-spectrum light-emitting diodes (LEDs) increasingly being favored because of their higher energy efficiency (*21*, *34*, *36*). The consequences of this shift are unknown, but it is predicted that white broad-spectrum LEDs have greater potential for ecosystem disruption, based on the visual sensitivities of many taxa, including nocturnal insects (*37*, *38*). These same studies suggest that narrower-spectrum lighting (e.g., sodium lamps, which emit mostly yellow light) may be less harmful to biological processes.

Here, we evaluated the impacts of nighttime lighting on wild caterpillars in southern England using a matched-pairs design, comparing habitat directly lit by established streetlights with carefully matched unlit habitat located nearby (≥60 m from the nearest streetlight). We took this approach because it provides insights into the long-term effects of real-world lighting intensities on wild insect populations. Practically, this approach also permits for much larger, spatially independent sample sizes that examine longer-term effects of lighting than manipulative field experiments (*39*), which are invariably more costly and tend to have more limited spatial replication. Both approaches have advantages and disadvantages, but we hope that our study acts to complement and enhance existing experimental findings (*40*).

We used moths as a proxy group for nocturnal insects more broadly (*26*). We focused on a relatively sedentary life stage (caterpillars), rather than adults, because this offers a clearer understanding of the impacts of ALAN at the population level. By sampling larval stages, we hope to demonstrate the effects where insects live and develop (and not simply where they happen to fly past). In addition, our approach avoids the use of light traps (the standard method of sampling moths), as these lead to biases when comparing assemblages from lit and unlit areas (*39*). The streetlights at two matched lit-unlit pairs of sites were divided between LED and high-pressure sodium (HPS) lamps plus two using older low-pressure sodium (LPS) technology. This allowed us to test both for an overall effect of lighting and any differences between narrow- and broad-spectrum lamps.

We sampled caterpillars along lit and unlit transects to test for a difference due to ALAN in local abundance and larval mass, a proxy for development. We used two sampling methods: hedgerow beating during the day (13 sites) and nighttime sweep netting of grass margins (15 sites). We hypothesized that caterpillar numbers would be lower on lit transects because of the multitude of negative impacts that are known from ALAN throughout moths life cycles (*26*). Separately, we installed LED and HPS experimental lighting rigs in field margins with no history of lighting to test our hypothesis that ALAN would disrupt the feeding behavior of nocturnal caterpillars. In all cases, we predicted that the effects would be most pronounced for broad-spectrum white LEDs, as physiological predictions suggest that these will be most disruptive for biological processes (*37*, *38*).

## RESULTS

**Fig. 1:**
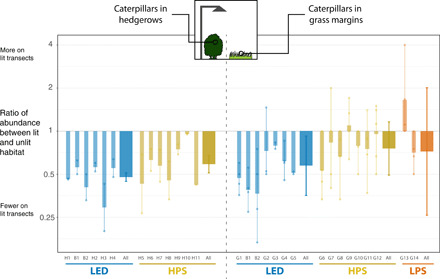
Paired differences in the abundance of caterpillars from hedgerows (left) and grass margins (right). The thick bars with 95% confidence intervals represent the overall treatment effect size from the GLMM. The narrow bars show the paired difference in caterpillar abundance at individual sites (number of lit caterpillars + 1)/(unlit caterpillars + 1), shown on a log_2_ scale). Each point shows the individual ratio for a single visit, and the solid bar gives the mean of these visits. Visits where no caterpillars were found on either the lit or unlit transects are not illustrated (but are included in the GLMM). Site codes indicate sites for hedgerows (“H”), grass margins (“G”), or both types of sampling methods (“B”). Further details on these field sites are contained in table S1 and fig. S1. Abbreviations used in the plot: LED, light-emitting diode; HPS, high-pressure sodium; and LPS, low-pressure sodium. Section S6 provides the spectral power distributions and estimated correlated color temperature (CCT) of the lights used as treatments.

Caterpillar abundance was substantially lower in habitat areas illuminated by streetlights. There were fewer caterpillars in lit hedgerows at all sites (overall effect on abundance = −47%, −52% for LED transects, and −41% for HPS; all *P* < 0.001, based on 1656 caterpillars beaten from hedgerows over 25 visits to 13 matched pairs of sites; Fig. 1). There were generally fewer caterpillars in grass margins on transects at almost all sites (overall effect on abundance = −33%, *P* = 0.01; −43%, *P* = 0.02 for LED; nonsignificant effects of −24% for HPS, *P* = 0.20; and −11% for LPS, *P* = 0.78; based on 822 caterpillars collected during 64 visits to 15 sites; Fig. 1).

**Fig. 2:**
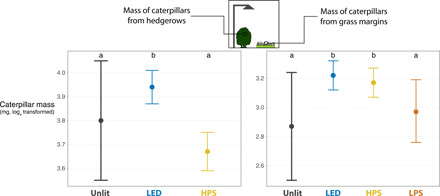
Estimates and SEs from the GLMMs for the mass of hedgerow caterpillars (left) and grass margin caterpillars (right) collected from field sites with long-term existing street lighting. Significant (*P* < 0.05) pairwise differences are shown using letters. The model includes random effects for site and caterpillar identity, hence why SEs may overlap despite statistically significant differences. Section S6 provides the spectral power distributions and estimated CCT of the lights used as treatments.

Moth caterpillars sampled from lit transects were typically heavier than those from unlit areas (Fig. 2), likely because ALAN heightened developmental rates. Tested with a generalized linear mixed-effect model (GLMM), including caterpillar morphotype to account for potential differences in the community composition, the effect was significant for LED (grass margins, *P* < 0.001; and hedgerows, *P* = 0.04), mixed for HPS (grass margins, *P* = 0.007; and hedgerows, *P* = 0.10), and nonsignificant for LPS (*P* = 0.60).

**Fig. 3:**
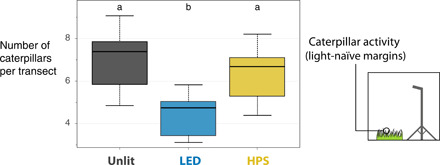
The plotted predictions from the GLMM for the short-term (1 to 2 hours) experiment in grass margins with no history of lighting. This was done to test whether using experimental lighting rigs prevented the normal behavior of nocturnal caterpillars, which is to climb up grass stems to feed. The horizontal line corresponds to the median, the boxes show the interquartile range, and the whiskers extend to the minimum and maximum data point. Significant (*P* < 0.05) pairwise differences are indicated with letters. Section S6 provides the spectral power distributions and estimated CCT of the lights used as treatments.

In a separate experiment, we erected lighting rigs along homogeneous, previously unlit grass field margins 1 hour before sunset. Sampling was conducted between 1 and 2 hours after dusk to test whether ALAN disrupted the normal feeding behavior of nocturnal caterpillars. Fewer caterpillars were sampled by sweep netting under white LED light compared to unlit (*n* = 9; effect size = −0.44; *P* = 0.03; Fig. 3). There was no statistically significant difference under HPS lights (*n* = 9; effect size = −0.10; *P* = 0.58).

## DISCUSSION

By focusing on a relatively sedentary life stage, our results provide strong evidence that street lighting negatively affects the local abundance of wild insect populations. The observed effects of −47% in hedgerows and −33% in grass margins were far greater than a previous study on adult macromoths (a −14% change after 5 years), which used experimental LED lighting, rather than real-world streetlights (*40*). Our results show that entire life cycles, not just single stages (e.g., conspicuous and mobile adult insects), should be considered to better understand the local impacts of biodiversity drivers on insect populations.

Our findings also reveal that the number of adult insects attracted to different lighting technologies may not serve as a suitable proxy for their ecological impact, as has often been previously assumed (*41*, *42*). For instance, a recent meta-analysis showed that LEDs tend to attract similar numbers of (or slightly fewer) moths than sodium lamps (*26*); thus, LEDs would be expected to be less damaging to moth populations. Yet, we found that the LEDs at our field sites had greater impacts than HPS lamps. This could suggest that flight-to-light behavior is not the principal mechanism via which moth populations are negatively affected by ALAN, although this hypothesis requires further confirmation and research.

From the several mechanisms that could drive the notable reduction in local caterpillar abundance [see review (*26*)], we suggest that decreased oviposition in lit areas is an important cause because moths in lit areas can have disrupted activity (*39*) and may lay fewer eggs (*30*). Indirect effects might also have added to the observed results. There could be increased top-down effects via niche expansion of diurnal predators, especially parasitoids (*43*, *44*). There might be bottom-up effects on host plant quality: HPS lighting negatively affects the development of *Apamea sordens* (a noctuid moth) by causing the food plant, grasses, to become physically tougher in lit areas (*45*).

Our study design used existing street lighting for treatments, so it does not represent a randomized, manipulative experiment. Our approach has benefits; for example, it allows for large spatial replication at a tiny fraction of the cost of constructing many artificial streetlights and also provides the ability to measure longer-term impacts. Our careful site pairing criteria (section S1) mean that we are confident that the notably large effect sizes resulted from street lighting. Although we cannot eliminate the possibility of unknown confounding factors influencing our results entirely, we are confident that differences between lit and unlit transects were not affected by dissimilarity in botanical composition (section S2), road verge management (section S1) and levels of urbanization surrounding the transects (section S3). Streetlights usually exist for one of two reasons: safety at junctions or proximity to urbanized areas. It is possible that lit transects, some of which were near junctions, might have experienced slightly elevated car noise, air pollution (e.g., NO*_x_*), or headlight glare (*46*), but we expect that the influence of such factors would have been minor compared to the effect of streetlights.

The guild hedgerow caterpillars appeared to be more adversely affected by ALAN compared with those in grass margins (Fig. 1). The higher lux values at hedgerow sites could be one explanation (with caterpillars in hedgerows being closer to the lighting than those in grass); however, the shade from hedgerow foliage might be expected to negate this. Adult mobility may be an important explanatory factor, with these hedgerows dominated by winter-active geometrids (which mostly have flightless females) and weak-flying micromoths. Conversely, the grass feeders were noctuid species, which are more mobile. Some have suggested that populations of less mobile insects would have greater sensitivity to ALAN (*47*, *48*). Different moth families are attracted to light to varying degrees (*49*), which may also contribute to this result. It is unclear how different families respond to the spectral outputs produced by the different types of streetlights in this study (LED and HPS) and even less is known about how responses differ throughout their life cycle (e.g., how various lighting spectra affects larval stages).

The finding of generally heavier caterpillars in lit areas (Fig. 2) is consistent with laboratory studies of two noctuid moth species, which demonstrated that stressed individuals increased developmental rates under ALAN (*45*, *50*). This finding seemingly contrasts with the result of our subsequent short-term experiment, which showed that the feeding behavior of caterpillars in grass margins was disrupted by LED lighting but not HPS lamps (Fig. 3). These caterpillars usually spend the day near the ground and climb grass stems at night to feed. This suggests that feeding behavior is readily disrupted by broad-spectrum light, which is more spectrally similar to daylight. So why would caterpillars, whose feeding behavior was most affected by light in our short-term experiment, also be the heaviest at the time of sampling at the roadside sites? Lighting attributes could have contributed to this difference. The brighter, bluer light source used for short-term experiment (25.3 lux, c. 5000 K; transect mean) is one explanation. The sites with existing streetlights had corresponding values that were lower and “warmer” (2.2 lux, 2700 to 4000 K; transect mean). See section S6 for details. Feeding was not prevented at the sites with streetlights, so heavier caterpillars could be due to intensified feeding but at atypical times of day or plant locations. It is possible that some form of adaptation or acclimation has occurred locally in areas with lighting [e.g., (*51*)], allowing nocturnal caterpillars to become active at night despite illumination. Caterpillars that were heavier in the lit section at the time of sampling may suggest advanced development under stress and investment in earlier pupation. This is predicted to have deleterious effects on adult fitness (*50*). However, even if earlier pupation did occur, it did not account for the observed differences in caterpillar abundance between lit and unlit transects (section S7).

How our results scale up to landscapes is critical to understanding the contribution of ALAN to insect declines. We do not know how far the impacts of lighting on caterpillars extend beyond the directly lit area, although this effect has been observed with moth pollination (*30*), so the extent of the spillover effects of lighting on species across their lifecycle is an urgent focus for further research. All our unlit transects were ≥60 m (median, 118 m) from the paired lit transects, and further investigation detected neither positive nor negative spillover effects in this study (section S4).

It is also important to consider how our results might scale up to entire regions or countries. By assuming all major roads have streetlights, one study estimated that streetlights affect 3.2% of the United Kingdom at >1 lux (*52*). Using spatial datasets that provided the actual distribution of streetlights, we estimate that 1.1% of the land area of the region enclosing our study sites is currently directly illuminated to this level once the area of road surfaces and urban land use (concrete surfaces, large buildings, etc.) is excluded (section S5). Suburban areas are frequently lit (15.5%), but only 0.23% of arable and 0.68% of broadleaved wood directly lit. Thus, we conclude that the effect of direct illumination by streetlights has probably been a minor contributor to long-term national moth declines to date; however, our results show that it can be a very substantial local factor. Given the ongoing expansion of ALAN, combined with our results, harmful impacts from outdoor lighting on long-term nocturnal insect populations may become more substantial in the future.

Even localized reductions in insect numbers could cause considerable cascading consequences for ecosystem functions and on other taxa. For instance, the caterpillar assemblage found in hedgerows in the spring forms an integral part of the diet of some songbird chicks (e.g., tits) (*14*, *53*, *54*). These bird species have a small foraging range (*55*–*57*) and, thus, are likely to be adversely affected by drops of up to 50% in the abundance of their prey that we found.

The ongoing shifts in streetlight technology, in particular the roll out of brighter types of light (typically white LEDs), are likely to be important for insects. Much of the population-level research examining the impacts of these changes in lighting has focused on the responses of vertebrate taxa, in particular bats [e.g., (*58*–*62*)], and has shown mixed results. Our work complements the studies carried out on bats by focusing on their prey (i.e., nocturnal insects). More research is needed to understand the effects of ALAN at the base of the food chain; and from this, indirect impacts of lighting on higher taxa through networks of ecological interactions can be examined.

Overall, we demonstrate how established streetlights have detrimental effects on local caterpillar assemblages. While further work is needed to unravel the relative importance of light pollution for insect population declines (especially compared to more pervasive threats such as habitat loss and climate change), our results show that ALAN acts as an important contributory driver for moth populations at the local scale, with ramifications for ecosystem processes including pollination and prey provision. With the ongoing increase in the extent and intensity of ALAN globally (*34*), urgent research is needed to understand how best to mitigate its effects on insects across life cycles. The impacts that we observed—on local abundance, development, and feeding behavior—were more pronounced for white LEDs compared to traditional sodium lamps (e.g., HPS lamps, yellow hues). This is worrying, given the current shifts in outdoor lighting technologies toward white LEDs (*21*, *34*). Yet, LEDs can be modified more easily than sodium lamps by adjusting their intensity (dimming) and spectral output (custom colors and filters) (*38*, *63*, *64*), offering the opportunity to minimize the negative impacts on insect populations, and linked ecosystem processes, at marginal costs.

## MATERIALS AND METHODS

### Field sites

We compared moth caterpillar communities at lit and unlit sections of sites within a matched-pairs design in two types of habitats: hedgerows and grass margins (which each used different sampling methods). Twenty-six pairs of sites were used, where a comparable linear section of both lit and unlit habitat was present. One additional site was a triplet (one unlit section and two sections lit with different streetlight types). All sites represented contiguous, linear strips of habitat, with lit and unlit sections separated by ≥60 m (median, 118 m; range, 60 to 527 m).

Potential pairs were detected by overlaying spatial datasets of streetlights covering the counties of Oxfordshire, Buckinghamshire, and Berkshire (southern England, UK) on satellite imagery to identify linear sections of habitat lit by at least one streetlight. Using Google Street View, “virtual site visits” were then made to more than 500 locations to identify whether contiguous and comparable habitat existed for lit and unlit transects. Of these, 153 locations were then visited in person to assess whether the matching criteria were met (section S1). This produced 26 pairs and one triplet where lit and unlit sections of habitat appeared identical, except for the presence of ALAN.

The streetlight treatments reflect the current lighting technologies used in the region. These were predominately LED and HPS, with two LPS sites (14 HPS transects, 11 LED transects, and 2 LPS transects). Spectral power distributions and estimated temperatures of the lighting are given in section S6. According to the data provided by the relevant local authority, the lit transects had been illuminated by the same lighting treatment type for at least 5 years (this was often much longer, with some lamps being in place for several decades), so any differences in moth communities represent long-term impacts. The lit sections of all study sites remained fully lit for the entire night; all field sites were visited at least once between 02:00 and 04:00 to confirm that part-night lighting or dimming was not in operation. The lit sites were often illuminated by streetlights on junctions or roundabouts in a rural setting, meaning the effects of ALAN were largely independent of urbanization, which can have deleterious impacts on moth communities (*65*). To ensure that we could robustly disentangle the effects of ALAN from other elements of urbanization, we conducted a GIS (geographic information system) analysis, which showed that the proportion of urbanization at various spatial scales was not a useful predictor of caterpillar abundance (section S3).

Light intensity was recorded using a lux meter (resolution, 0.1 lux; Andoer HP-881C) at five evenly spaced points along each transect. This was done on overcast nights or during the new moon. Readings were taken directly upwards at the height likely to be experienced by the caterpillars: 1.25 m for hedgerows and 0.25 m for grass margins. Lit hedgerow sites ranged from a transect average of 1.42 to 15.84 lux (overall mean, 5.7 lux), while lit mean grass transects varied between 0.18 and 7.14 lux (overall mean, 2.2 lux). Within each site type, the mean lux values of sodium and LED transects were not statistically different for either hedgerows [independent *t* test, *t*(7.6) = −0.97; *P* = 0.34] or grass margins [*t*(13.9) = −0.2, *P* = 0.85]. All unlit transects were estimated to be <0.01 lux on an overcast night (and all measured the minimum reading of 0.1 lux on the light meter).

An important caveat is that lux is based on human vision, and thus potentially ecologically relevant spectral information can be omitted when using this unit (*66*). Identical measurements of lux may not correspond to the same illumination as perceived by a caterpillar. Despite these shortcomings, lux is the SI unit for light intensity and continues to be used by ecologists and urban planners alike due to its convenience. Thirteen pairs of sites had comparable sections of hedgerow for beating and 15 had suitable strips of grass margin for sweep net sampling (two sites were used for both types of sampling).

### Caterpillar sampling

Two sampling methods were used to test the responses of the two feeding guilds of moth caterpillar communities at lit and unlit transects: hedgerow beating in spring for species feeding on deciduous trees and hedges (largely winter-flying geometrids) and sweep net sampling in winter for overwintering noctuid species feeding nocturnally on grasses (the adults largely fly in the autumn). Hedgerows were sampled during the day once in mid-May 2019 and once in mid-April in 2020. These dates correspond to the end and start, respectively, of the prime season for moth caterpillars feeding on the spring flush of foliage (largely winter-flying geometrids). These two specific periods were chosen so that any phenological artifacts on abundance arising from ALAN could be removed. In the late spring sampling, caterpillars were the fourth or final instar, while in the early spring visit, caterpillars were the first or second instar. This means that we sampled at the start, and at the end of the time, this group (spring flush feeders) spends as a caterpillar so phenological effects were negated (section S7). One site had been developed into housing between the two visits so it was not sampled in 2020. Beating was conducted at three points along each transect, which were 14 m long. The dominant plant species of the hedgerow was recorded and was typically the same at a paired site (e.g., six *Crataegus*-dominated sampling points). Where this was not possible, the dominant species composition was kept constant within a pair (e.g., two unlit *Crataegus*-dominated points and one unlit *Acer campestre* paired with two lit *Crataegus* and one lit *A. campestre*). Beating used two methods: drainpipes for box-shaped hedges (eight sites) and a beating tray (five sites). Three 2 m lengths of half drainpipe (width, 11.2 cm) were inserted at the base of the hedge lying next to each other perpendicular to the hedge direction [a modification of methods by (*67*, *68*)], while a traditional beating tray (dimensions, 110 cm by 86 cm; Watkins & Doncaster) was used at sites where there was overhanging vegetation. In both cases, the vegetation was struck hard five times with a metal pole to dislodge caterpillars.

The second method was sweep netting grass margins for overwintering noctuid caterpillars, which climb up grass stems to feed at night. Transects were established either on the roadside (road verges) or the field side (agricultural margins), depending on where comparable habitat was available. Botanical surveys were conducted during June 2019, and Analyses of Similarities showed that for each site, the plant community of lit and unlit transects were indistinguishable (section S2). Caterpillar sampling took place on mild nights (forecasted minimum temperature ≥6°C) from November 2018 to April 2019 and was done at least 1 hour after sunset between 21:00 and 06:30. Transects were walked at a consistent pace while making brisk sweeps in a continuous figure-eight motion with a sweeping net (diameter, 50 cm; pentagon-shaped frame; Watkins & Doncaster). The number of caterpillars recorded per transect was relatively low (mean, 6.9) so sites were visited several times over the season; most sites were sampled four times. At one site, new streetlights were installed in the previously unlit section during the study period, so this site was only visited twice. Both transects in a pair were sampled an equal number of times so, while there was variation between sites, the statistical comparison between lit and unlit transects is wholly unaffected by this variation in the number of visits. Transects were marked with plastic markers on the first visit and were typically 14 m long but, at some sites, were longer if there was enough comparable habitat available across both sections of the pair (transect length was always kept the same between lit and unlit sections). Sampling of the lit and unlit sections was separated by a maximum of 10 min. The time of sampling (later converted to minutes past sunset) and the temperature (according to the external car thermometer) were recorded.

All caterpillars collected from the grass strips (*n* = 826) and the late spring hedgerow sampling (*n* = 1021) were retained (early spring caterpillars from 2020 were not kept due to the lack of laboratory access because of coronavirus pandemic restrictions). Provisional identifications were assigned by the lead author using prior knowledge, (*69*), and www.ukleps.org. Hedgerow caterpillars were predominantly winter-flying geometrids (largely *Operophtera* spp. as well as *Epirrita* spp., *Agriopis* spp., *Erannis defoliaria*, and *Phigalia pilosaria*) and also summer-flying micromoths (*Tortricidae* spp., *Acrobasis* sp., and *Ypsolopha* sp.). These were grouped into 20 “taxonomic units” (which includes species, genera, family-level determinations, and “unknowns”). Grass margin caterpillars were overwhelmingly overwintering, grass-feeding noctuids (predominately, *Xestia xanthographa/Xestia sexstrigata*; also, *Mesapamea* spp., *Noctua pronuba*, and *Phlogophora meticulosa*). Thirty-four day-flying lepidopteran caterpillars were recorded (*Nymphalidae* and *Zygaenidae*). These were included in the analyses because day-flying species with nocturnal larval stages might still be adversely affected by ALAN (*26*), and the sample size was too low to test for a difference. Grass margins produced 16 taxonomic units (as defined above for the hedgerow guild).

All caterpillars were weighed using a digital analytical balance (resolution, 0.0001 g). The mean mass of caterpillars from grass margins was 98 mg (range, 0.07 to 2240 mg), and the mean mass of hedgerow caterpillars was 43 mg (range, 0.2 to 866 mg).

### Short-term experiment on caterpillar feeding behavior using lighting rigs

Two sites with no history of lighting were selected for a short-term experiment on the impacts of ALAN on the feeding behavior of nocturnal caterpillars. These were arable sites with long (550 and 320 m), homogenous strips of grass margin, where 27 transects of 14 m were established. These transects were measured with a click wheel and marked on the first visit. Four-meter-high lighting rigs fitted with either LED or HPS lights (imitating a residential streetlight) were erected at the midpoint of some transects, determined haphazardly before sampling to ensure all three active transects (LED, HPS, and the unlit control) on a given night were separated by at least 60 m. See section S6 for spectral outputs and temperatures on the lights used in this experiment. See (*29*) for additional details on the intensity outputs. Sampling was conducted between late January and mid-February 2020 using sweep netting on nine visits, with three transects typically being sampled on a given night. Mild nights (forecasted minimum temperature of >6°C) were chosen as they were expected to have higher levels of caterpillar activity. Lighting rigs were installed during the afternoon and switched on 1 hour before sunset. To give the nocturnal caterpillars time to become active, sweep netting occurred between 60 and 120 min after sunset (mean, 89 min); sunset time was taken from www.timeanddate.com. On a sampling night, all transects were sampled within a 5- to 10-min period and the treatment order varied haphazardly according to the location of the lighting rigs along the margin. The caterpillar assemblages were comparable to those found at the main study grass margin sites: overwintering grass-feeding noctuids, predominately *X. xanthographa/X. sexstrigata*. Lighting rigs were powered by petrol generators, which were positioned perpendicularly to the field margin at a distance of 50 m from the transect midpoint to remove any potential impacts on caterpillar activity arising from generator noise, vibrations, or fumes.

### Data analysis

GLMMs were used to examine differences in caterpillar abundance between lit and unlit sections. Models were constructed using the lmer package (*70*) in R (*71*). For both hedgerows and grass margins, the GLMM included a random effect for site (intercept only). Models were run with treatment as binary (unlit and lit) to estimate the overall effect of light and also with treatment as a categorical variable (unlit, LED, HPS, and LPS) so we could also test for varying impacts of different lighting technologies. The hedgerow models contained only year and treatment (unlit, lit/unlit, HPS, or LED) as fixed effects. The dominant hedgerow plant species and the hedgerow height were not important predictors of caterpillar abundance, so these variables were not included in the final model. The grass margin models included treatment (unlit, lit/unlit, LPS, HPS, or LED), additional fixed effects for the number of days since the start of sampling and minutes past sunset (these two variables were rescaled by dividing by 100), and an offset for transect length. The offset was calculated with the typical transect length (14 m) being assigned an offset value of “1” and longer lengths scaled accordingly (i.e., a 20-m transect would have an offset value of 1.4). Temperature was not a useful predictor of abundance, so it was not included in the final model. The models used a negative binomial distribution as the data were counts, which were overdispersed. Throughout, all models were carefully examined to ensure the key assumptions were met.

To assess changes in the rate of development due to ALAN (via a proxy and body mass), we used a GLMM to test the effect of log-transformed body mass against lighting treatment while also taking account of days since the start of sampling and including two random effects (intercept only): sampling visit and caterpillar identity (the latter to account for any differences in caterpillar assemblages arising from lighting treatments).

The short-term experimental data using lighting rigs were also analyzed using a GLMM model. This included the number of caterpillars as a response variable, treatment as a fixed effect (unlit, LED, and HPS), and a random intercept effect for sampling night (to account for possible differences in caterpillar activity on different nights that could arise from factors including site, moon phase, and weather). A Poisson error distribution was used, as these data were not overdispersed.

## SUPPLEMENTARY MATERIALS

Supplementary material for this article is available at http://advances.sciencemag.org/cgi/content/full/7/35/eabi8322/DC1
